# Insulin Resistance in the Offspring of Parents with Type 2 Diabetes

**DOI:** 10.1371/journal.pmed.0020289

**Published:** 2005-09-27

**Authors:** Anton J. M Wagenmakers

## Abstract

Wagenmakers discusses the paper by Petersen and colleagues on insulin resistance in young lean individuals and its association with reduced phosphate transport into muscle cells and impaired mitochondrial energy generation in muscle.

The global epidemic of type 2 diabetes is a pressing public health concern associated with a rapidly growing socioeconomic burden. Insulin resistance (IR) is an early event in the pathogenesis of type 2 diabetes. IR is characterised by a reduced ability of insulin to stimulate glucose uptake in skeletal muscle and by hyperglycaemia (high blood glucose concentration), at first only in periods following meal ingestion but later also in the overnight-fasted state.

In patients with type 2 diabetes, IR is progressive and after several years often leads to the development of secondary diabetes symptoms (including hypertriglyceridaemia, obesity, and pathology of the macro- and microvasculature). Eventually (after more than ten years) severe medical complications may develop, including retinopathy, neuropathy, tissue necrosis in the extremities, renal failure, and cardiomyopathy.

A progressive reduction over the last few decades of daily engagement in physically demanding activities and the recent growth in unbalanced diets are generally considered to be the primary causes of the dramatic rise in type 2 diabetes [1]. A genetic predisposition also runs in families and populations. In this month's *PLoS Medicine*, Kitt Petersen and colleagues [2] report new information on the early events in the underlying pathogenic mechanism that leads to the development of IR.

## The Study's Findings

The authors investigated young, lean offspring with IR of parents with type 2 diabetes [2]. The reason to select offspring with IR is that a metabolic defect observed in this group is likely to be an early event of genetic origin and, therefore, is potentially a primary cause of the subsequent development of type 2 diabetes.

The offspring with IR were studied during a hyperinsulinaemic–euglycaemic clamp. This test is traditionally used to diagnose IR. The test measures the ability of insulin to stimulate the clearance of glucose from the blood during a simultaneous infusion of insulin in supraphysiological quantities and of glucose in quantities sufficient to maintain the glucose concentration at a normal physiological level. Petersen and colleagues found that infusion of insulin increased the turnover rate of adenosine triphosphate (ATP) in the skeletal muscle of control participants by 90%, while only a 5% increase (nonsignificant) was seen in offspring with IR. The increase in ATP turnover in the control participants means that the metabolic rate (energy expenditure) of the muscle goes up during the clamp study. In an earlier study by the same group of researchers [3], offspring with IR also were observed to have 30% lower rates of muscle ATP turnover in the overnight-fasted state.

## What Do These Findings Mean?

**Figure 1:**
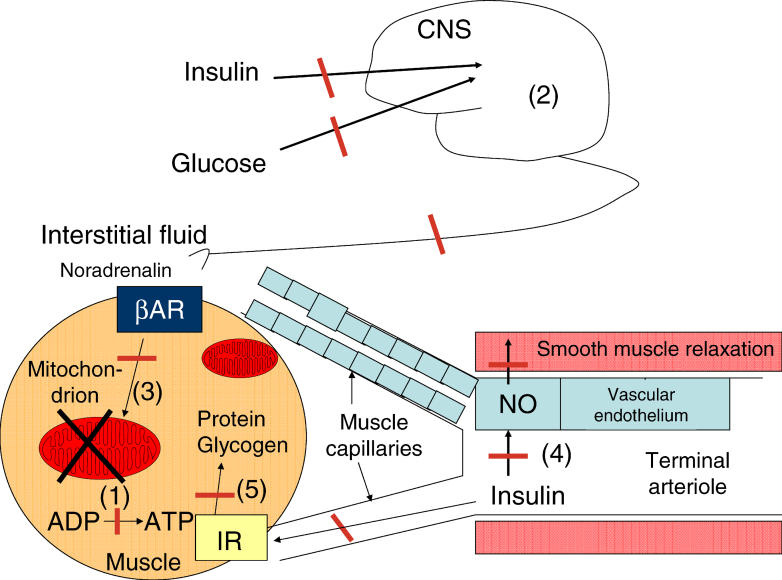
Potential Mechanisms Leading to Failure of Insulin to Stimulate Muscle ATP Turnover during a Hyperinsulinaemic–Euglycaemic Clamp in Offspring with IR The potential mechanisms are (1) a general mitochondrial dysfunction, reducing ATP production; (2) an impairment in the central nervous system (CNS) in the glucose- or insulin-induced excitation of muscle efferents, leading to reduced β-adrenergic activation of the muscle; (3) a reduced increase in mitochondrial ATP synthesis in response to activation of the β-adrenergic receptor (βAR); (4) a defect in insulin-induced opening of the terminal arterioles controlling blood flow through muscle fibre capillaries and, thus, preventing increases in the insulin concentration in the interstitial fluid and in binding of insulin to the insulin receptor (IR) in the muscle membrane; and (5) a molecular defect in the insulin signalling cascade in the muscle, leading to reduction in the insulin-induced stimulation of muscle glucose uptake, glycogen synthesis, and protein synthesis. ADP, adenosine diphosphate; NO, nitric oxide.

ATP in resting fasted muscles is only produced for purposes of cell maintenance and survival functions (for example, maintenance of sodium and potassium gradients, amino acid gradients, protein synthesis rates, and functional organelles and membranes). Hence, Petersen et al.'s observations suggest that either the basal energy requirement is reduced in muscles of individuals with IR (potentially at the expense of the maintenance of cell functions) or the major control systems for mitochondrial respiration (simultaneous ATP synthesis and consumption) are not properly working (Figure 1).

Petersen and colleagues favour the first explanation. They suggest that their combined observations point to a general mitochondrial dysfunction that impairs the ability of the mitochondria to synthesise ATP and oxidise fatty acids (FAs) at the normal resting rate, both in the basal overnight-fasted condition and after stimulation by insulin [2,3]. They also suggest that it is this mitochondrial dysfunction that causes IR.

The mitochondrial dysfunction is hypothesised to lead to a reduced ability to oxidise FAs and to the accumulation of triglycerides and FA metabolites (fatty AcylCoA, diacylglycerols, and ceramides). Such accumulation of triglycerides and FA metabolites has been repeatedly observed, both in skeletal muscle of obese individuals [4,5] with a reduced insulin sensitivity and in the muscle of healthy individuals given IR by the infusion of lipid emulsions and heparin [6]. These FA metabolites have been linked to the development of IR via a mechanism involving activation of protein kinase C and phosphorylation of the insulin receptor and IRS-1 at serine and threonine amino acid residues. Phosphorylation at these wrong amino acid residues prevents insulin-induced tyrosine phosphorylation of the insulin receptor and IRS-1, and prevents activation of the insulin signalling cascade; therefore, this mechanism is presently regarded as the primary cause of IR at the molecular level in the muscle [4–6].

Regular exercise and training should be considered interventions to correct the reduction in insulin-induced muscle ATP turnover.

But there is a problem with the assumption that a general mitochondrial dysfunction reduces both the basal and insulin-stimulated ATP production. In the muscle of both healthy control participants and patients with IR and type 2 diabetes, there is a large overcapacity in the ATP production capacity of skeletal muscle, allowing 5- to 20-fold increases in ATP turnover during exercise. Defects in resting mitochondrial ATP production, as occur in the muscle of patients with metabolic myopathies, lead to major reductions in the resting creatine phosphate/ATP ratio, and to a parallel increase in muscle lactate production as a consequence of a compensatory increase in the glycolytic ATP production. However, the offspring with IR in the previous studies by Petersen et al. [2,3] had normal resting creatine phosphate/ATP ratios and no change in muscle pH as a consequence of excessive lactate production. These findings seem to argue against the hypothesis that there is a general mitochondrial defect in offspring with IR and patients with type 2 diabetes.

## Alternative Explanations for the Findings

An important open question concerns the mechanism by which insulin would increase the resting muscle ATP turnover rate in the control participants. Total energy expenditure in human tissues can be roughly broken down in three components: (1) obligatory energy expenditure required to perform cell maintenance and survival functions and maintain cell and body temperatures at 37 °C, (2) adaptive energy expenditure induced by nutrient ingestion, and (3) energy expenditure required to perform muscle contractions and physical activity [7–9]. Total energy expenditure is the sum of the energy required to perform all cellular and organ functions, plus heat production.

One possibility is that the increase in muscle ATP turnover, induced by insulin during the clamp, is caused by the well-known thermogenic effects of glucose and insulin [7–9]. Muscle glycogen synthesis and protein synthesis are energy-requiring metabolic processes, and both are stimulated when insulin binds to the insulin receptor in the muscle membrane. The stimulation of glycogen and protein synthesis leads to a need to increase muscle ATP turnover. As the efficiency of mitochondrial respiration is only 40%, increases in the rate of these metabolic reactions by definition contribute to heat production and the thermogenic effect of food ingestion. Therefore, insulin will increase muscle ATP production during the hyperinsulinaemic–euglycaemic clamp in healthy muscles in comparison to the basal state, while smaller increases in glycogen synthesis, in protein synthesis, and, therefore, in ATP production will occur in offspring with IR. This alternative mechanism explains the findings observed in Petersen et al. [2], but does not implicate that there is a pre-existent mitochondrial dysfunction.

The autonomic nervous system is known to modulate the thermogenic effect of glucose by activating small efferent nerves that end in the interstitium of skeletal muscle (the fluid that surrounds the muscle fibres). The nerve endings produce noradrenalin, which activates β-adrenoreceptors in the muscle membrane, and this leads to an increase in mitochondrial ATP production. The part of glucose-induced thermogenesis that is eliminated by β-adrenergic antagonists has been called “facultative thermogenesis” and is assumed to take place at least in part in skeletal muscle [9]. It has also been suggested [9] that insulin, via unidentified receptors, most probably located in the central nervous system, may stimulate muscle sympathetic nerve activity and facultative thermogenesis. The thermogenic effect of insulin and carbohydrates has been shown to be reduced in obese and insulin-resistant individuals [8,9]; therefore, an impairment in the mechanism leading to facultative thermogenesis may also explain a part of the reduction in insulin-stimulated muscle ATP synthesis observed in offspring with IR.

Recently, it has also been shown that insulin infusions lead to increases in blood flow through capillaries that surround the skeletal muscle fibres, both in healthy humans and rats [10–12]. The mechanism involves the binding of insulin to the insulin receptor on the endothelial cell layer that covers the luminar wall of the terminal arterioles that control blood flow through the muscle capillaries (Figure 1). This binding leads to activation of the insulin signalling cascade in the endothelial cells and to nitric oxide production [11–13]. Nitric oxide, a muscle relaxant, then diffuses to the smooth-muscle cell layer and leads to relaxation of the sphincter muscle, dilation of the terminal arterioles, and recruitment of muscle capillaries (Figure 1). The opening of the muscle capillaries in healthy control rats precedes the insulin-induced increase in glucose uptake, and the increase in glucose uptake can be prevented by prior infusion of nitric oxide synthase inhibitors. These observations suggest that insulin first recruits muscle capillaries before it can reach the insulin receptor in the muscle membrane and stimulate muscle glucose uptake, glycogen and protein synthesis, and ATP production. Severe defects do exist in this insulin-induced opening mechanism in obese insulin-resistant Zucker rats [14]. Although failure of the insulin-induced recruitment of muscle capillaries has not yet been shown to exist in humans with IR, it could also explain the reduced insulin-induced increase in muscle ATP synthesis rates in the offspring with IR observed by Petersen et al. [2], again without pointing to a mitochondrial defect or dysfunction.

## The Study's Clinical Implications

Failure of insulin to stimulate muscle ATP production in offspring with IR may have multiple causes. A general mitochondrial dysfunction, as proposed by Petersen and colleagues, is one possibility, but the failure of insulin to (1) stimulate the insulin signalling cascade in muscle, (2) stimulate central thermogenic-control mechanisms of mitochondrial respiration, and (3) recruit muscle fibre capillaries are other potential mechanisms.

The basal observation that glucose and insulin do not stimulate muscle ATP production and thermogenesis in individuals with IR is clinically highly relevant, as it may explain the weight maintenance problems that people with IR experience. When there is a gradual reduction in the basal and insulin-induced energy expenditure at the muscle level during the development of type 2 diabetes, food intake should be reduced in proportion to the lower ATP need of the muscles. Failure to correct for the lower muscle energy requirement will lead to a positive energy balance and to weight gain. The data in Petersen and colleagues' study [2] also seem to suggest that the relative increase in energy expenditure by glucose and insulin is larger (90%) at the level of the muscle than at the level of the whole body (the whole-body thermogenic effect of orally ingested carbohydrates is maximally about 10%–15% [8,9]). A gradual disappearance of this large energy component in individuals with IR will lead to a substantially lower calorie and nutrient requirement.

Regular exercise and training should be considered interventions to correct the reduction in insulin-induced muscle ATP turnover. Endurance exercise performed three to four times per week may lead to more than 5-fold increases in the mitochondrial density (concentration) of a previously sedentary muscle [15], and will increase the ATP generating capacity. Both endurance and resistance exercise increase insulin sensitivity at the molecular level in the muscle, and they have also been suggested to increase the sensitivity of adrenergic control in both skeletal muscle and adipose tissue [15]. Exercise and training open muscle capillaries and increase glucose uptake in skeletal muscle by contraction-induced mechanisms that are independent of insulin action [12,14]. The measurement of muscle ATP turnover with magnetic resonance spectroscopy, as used in Petersen et al. [2], seems to be an ideal noninvasive method to investigate one critically important question: can changes towards a more active lifestyle reverse the observed reduction in insulin-induced muscle ATP turnover in the offspring with IR, and, in parallel, restore insulin sensitivity of muscle and precapillary arterioles and delay or prevent the later development of type 2 diabetes that was present in the parents?

## Abbreviations

ATP: adenosine triphosphate
FA: fatty acid
IR: insulin resistance
